# Brain networks activated when aggravating baseline chronic pain of an individual with new daily persistent headache: a case study

**DOI:** 10.1186/s12883-025-04572-z

**Published:** 2025-12-26

**Authors:** James W. Lewis, Katerina Pawlowski, Max Hurley, Tyler McGaughey, Yuen Man Tze, Molly Summers, Shabrina Jarrell, Richard Nolan, Biff Alexander, Lauren E. Rentz, Michelle M Coleman, Sam Salmassi, Gang Chen, David Watson, Julie Brefczynski-Lewis

**Affiliations:** 1 Department of Neuroscience, Blanchett Rockefeller Neuroscience Institute, West Virginia University, Morgantown, WV 26506 USA; 2 Department of Neurology, West Virginia University, Morgantown, WV 26506 USA; 3 West Virginia Clinical and Translational Science Institute, Department of Neuroradiology, Morgantown, WV 26506 USA; 4 Scientific and Statistical Computing Core, NIMH, National Institutes of Health, Bethesda, MD 20892 USA

**Keywords:** MRI, Functional connectivity, Deep pressure stimulation, Neuropathic pain, Causality, Central mechanisms, Chronic daily headache, Touch

## Abstract

In this study we examined neuronal mechanisms associated with chronic headache pain by recruiting a participant diagnosed with New Daily Persistent Headache (NDPH), who had the reliable characteristic that his baseline chronic pain could be immediately and consistently modulated (partially relieved or aggravated) by applying deep pressure to specific locations along his face and head. We sought to map brain regions responsive to modulations of his baseline pain using functional magnetic resonance imaging (fMRI). We constructed customized MRI-compatible 3D-printed face masks and skull plates affixed with syringe plungers that could apply and remove deep pressure to discrete craniofacial locations. Using On/Off block paradigms, we collected fMRI data across nine sessions while pressing on locations that either increased, decreased, or had no effect on his chronic pain. Although displacement artifacts precluded use of relief point data, we did reveal five brain regions that showed significantly increased responses when stimulating locations that aggravated his baseline headache pain, including the right anterior insula, bilateral inferior parietal lobule (IPL) foci, plus bilateral cerebellar regions (lobule VIIIb). Using structural vector autoregression (Granger causality), resting-state functional connectivity MRI analyses of the above somatosensory-related aggravated pain network further revealed effective connectivity (positive effect paths) from the left IPL to the right anterior insula, and these two regions had positive effect paths to the right IPL. This cortical circuit was coupled with the cerebellar foci plus the participant’s anatomically derived periaqueductal gray (PAG) region. Moreover, on days when the participant had greater degrees of baseline chronic headache pain the right IPL and PAG exhibited negative effect paths on the left IPL and left cerebellum, respectively, thereby revealing additional psychophysiological attributes of this circuit. Together, these results not only identified candidate targets for patient-customized neuromodulation therapies but also revealed a novel testable circuit model regarding potential mechanisms underlying one form of neuropathic pain perception.

## Introduction

**Fig. 1:**
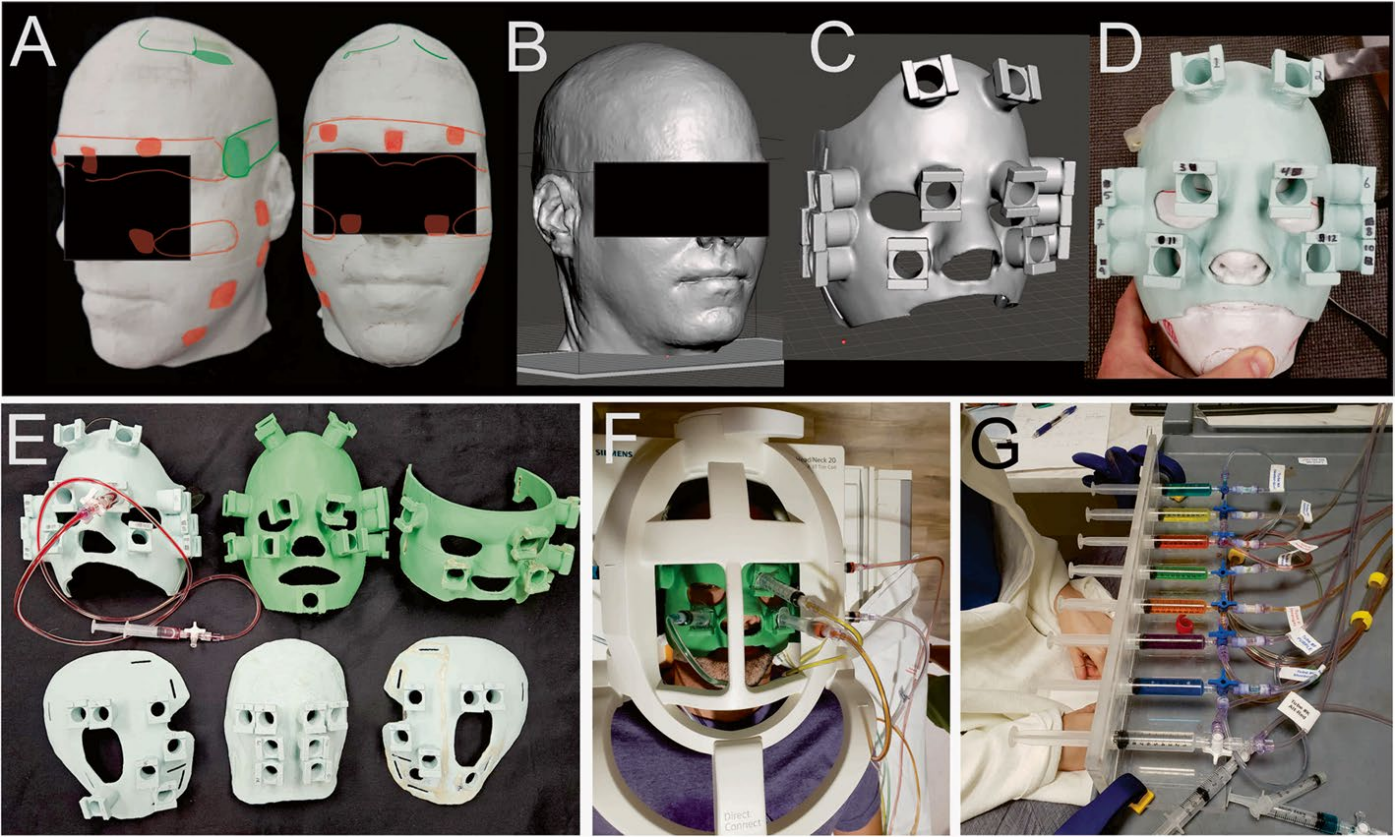
**A** Three-dimensional (3D) model of the headache participant’s head illustrating patient-reported locations where applying constant deep pressure would either immediately provide some relief (green, e.g., along his temples and scalp), aggravate his baseline chronic pain (red; mostly on face), or have no effect (white). (**B**-**C**) Example 3D rendering of the participant’s head and of a face mask customized to fit his face together with housings for inverted syringes as plungers. (**D**) 3D-printed face mask (depicted in panel C) using polylactic acid (PLA) filament. (**E**) The collection of face masks and head plates used over the course of nine fMRI scanning sessions. (**F**) The headache participant wearing a mask while lying in the 20-channel MRI headcoil. (**G**) Organization of the hydraulic plunger system, including eight plunger tube units comprised of syringe plungers, Luer-Lok adapters and tubes filled with differently colored water

Debilitating chronic headache pain disorders are largely “invisible” from a neuroimaging perspective, being very difficult to verify clinically independent of patient report, creating challenges for efforts in research aimed toward identifying objective measures of the underlying neuropathologies. The slow time course of headache pain onset and offset (hours or days) generally prohibit traditional individual-level neuroimaging approaches that require numerous repetitive measures between conditions (i.e., pain versus less or no pain), in that one cannot turn chronic headache pain on and off repeatedly. Notwithstanding, we were able to recruit a headache participant with a form of chronic pain, termed New Daily Persistent Headache (NDPH), who exhibited the reliable characteristic that applying constant deep pressure to specific regions on his face and head would immediately and transiently either provide partial relief (Fig. 1A, at green colored locations) or aggravate (at red colored locations) his baseline chronic pain. These effects were reportedly stable for over a decade; wherein, for instance, wearing a tightly fitting baseball cap or tight head band provided partial relief throughout the day (and night). Given the idiosyncratic nature of being able to rapidly modulate this headache participant’s baseline chronic headache pain, we sought to use functional magnetic resonance imaging (fMRI) On/Off block paradigms to map his brain in response to pain modulation. To achieve this, we created a series of customized MR-compatible 3D-printed face masks and skull plates affixed with inverted hydraulic plunger housings (Fig. 1B-G) that would allow the application and removal of deep pressure stimulation to specific locations.

One goal in brain mapping of pain networks, especially pain conditions resilient to pharmaceutical interventions, is to identify circuits that may be modified through neuromodulation. A vast literature on human and animal pain research has revealed numerous different cortical, subcortical, and associative brain regions that are activated during painful stimulation, which has been reviewed elsewhere [1–3]. With so much complexity relative to other sensory systems, such as dependence on the category of pain, definitions used to describe pain, different intensity levels or qualities, etc., some suggest that brain networks mediating pain perception may best be regarded as a pain matrix [4], often including somatosensory, insular, prefrontal, and anterior cingulate territories [5–7], plus subcortical regions such as the thalamus, basal ganglia, cerebellum, and periaqueductal gray, among others [8, 9]. With regard to brain mapping of pain networks in humans, headache pain research is typically divided into respective clinical categories (e.g. tension, migraine, cluster, nummular, etc.) and further divided into acute versus chronic pain [10, 11]: The research field of chronic headache pain (having headaches on 15 or more days per month for at least three months) is germane to the present case study, with our chronic headache participant being diagnosed with NDPH.

Though fairly rare, NDPH is a category of headache with a unique feature in that the chronification (“chronicity”) of pain is effectively instant, occurring one day that is typically remembered by the patient, and thus a hallmark of this disorder [12–16]. Other categories of chronic headache typically have a long history of graded effects and possible neural network adaptations with intermittent network states over time, wherein the unpleasant intensity of pain eventually leads to secondary pain affect states over the course of months or years [1, 9, 17, 18]. This drawn-out time course for individuals with more traditional forms of headache pain chronification may consequently be relatively difficult to track or measure through functional neuroimaging. In contrast, the immediate onset of NDPH chronic headache in one day does not appear to follow this classical sequential model of pain affect. Consequently, the mechanistic study of NDPH headache through neuroimaging techniques has a unique research advantage in that there is effectively a “switch” in an individual’s brain that suddenly turns “on” leading to a chronic headache mode.

Group-level structural neuroimaging studies of NDPH have been reported [19, 20], but largely remain unremarkable for clinical purposes at an individual patient level [21]. Additionally, NDPH is one of the most treatment refractory primary headache types [22–24], and migrainous or tension type pharmaceutical treatments are usually only partially effective or ineffective [25, 26]. Thus, more rigorous investigations of the neuronal mechanisms underlying NDPH has been warranted to facilitate non-pharmaceutical intervention approaches, such as electromagnetic neuromodulation of specific brain targets, as has been conducted in other areas of pain research [27–33].

Assuming that fMRI mapping of baseline chronic pain modulations would reveal brain regions of interest (ROIs) critically related to pain relief or aggravation, we further sought to collect multiple resting-state functional connectivity MRI (rsfMRI) datasets over the course of several months. Over this time span, the headache participant’s baseline chronic pain severity would fluctuate and thus be amenable to study of state-dependent effects. Analyses of these resting state datasets would, in principle, allow identification of both functional and effective connectivity characteristics (i.e. using Granger causality techniques) to independently assess potential circuit dynamics between any fMRI-derived brain regions (or “hubs”) as a function of our headache participant’s baseline chronic pain, which has been a basic approach used in earlier rsfMRI studies [20, 34–40].

Our first hypothesis was that deep pressure applied to the face and head locations that cause immediate, transient partial relief from this headache participant’s baseline NDPH chronic pain (“relief points”), relative to “sham” conditions that do not modulate his baseline pain, would lead to significant differences in brain activation patterns (presumably any of the numerous reported pain matrix territories) reflective of a network mediating the perception of pain relief. Conversely, our second hypothesis was that deep pressure at locations that would aggravate his baseline chronic pain (“aggravation points”), relative to sham conditions, would lead to significant activation of a different set of brain regions reflective of a network mediating the perception of aggravated chronic pain. Revealing specific brain regions responsive to baseline pain modulation would serve to (A) derive objective neuronal network measures of this participant's otherwise invisible chronic headache pain, and (B) identify individualized idiosyncratic (or possibly generalizable) brain ROIs that could be targeted in future studies using neuromodulation therapies.

## Materials and methods

### Participants

We recruited a chronic headache participant who had been diagnosed with New Daily Persistent Headache (NDPH) for neuroimaging visits over a span of 3 years (male, initially 41 years old). He suffered a sudden onset of intense headache pain he rated as a “9 out of 10” on a verbal report of pain [41], and remembers vividly the exact date of onset. He sought health care from multiple providers across the United States for twelve years prior to the present study. Over that span of time, he had been prescribed various therapies including standard migraine prevention and acute pharmaceutical options, onabotulinumtoxin injections, various trigger point injections, and peripheral nerve blocks. Gradual reduction in his baseline chronic pain was achieved but had yet to manage his persistent headache pain adequately or fully. A clinical anatomical screening revealed a cyst at the back of his throat; antecedent infectious prodromes and sinusitis can sometimes mimic NDPH [13, 42]. However, a procedure to address this over a decade ago did not rid his headache, and thus there remained no clear etiology. The participant’s headache type met criteria for NDPH as described in the International Classification of Headache Disorders (3rd ed. Section 4.10) [43]. During the time of the fMRI recordings (2019 through 2022), he was taking Erenumab (Amovig), a monoclonal antibody drug that is designed to block pain via antagonism of calcitonin gene-related peptide (CGRP). He was in otherwise good health and gainfully employed in his profession.

Handedness was assessed based on 12 questions from the Edinburgh Handedness Inventory [44]. This resulted in a handedness quotient of −91 (range −100 to + 100), and no family history of left-handedness. We mapped his brain responses to spoken language (see below), which did not reveal any atypical language lateralization. He was thus considered to be strongly right-handed and left-lateralized for language processing.

Based on scheduling convenience, our headache participant came to our site roughly once every two months, and on nine of the separate visits completed an experimental session of baseline headache pain modulation mapping using functional magnetic resonance imaging (fMRI). Deep pressure applied to distinct craniofacial regions would consistently either partially relieve his baseline chronic pain (Fig. 1A, at green colored face/head regions demarcated by the headache participant), aggravate his baseline chronic pain (red), or would have no effect on his chronic pain (white). This fMRI brain mapping included a series of nine experiments for which MRI-compatible face masks and skull plates were constructed (Fig. 1, Table 1) fitted with inverted plastic syringes (5 ml, 6 ml, or 10 ml) as hydraulic plungers with interconnected tubes (detailed below) to allow the application and withdrawal of deep pressure to various craniofacial pressure points.

One healthy control participant (male, aged 24; right-handed, based on Edinburgh Handedness Inventory) with no history of headache or chronic pain was recruited to perform a variety of MR equipment tests and fMRI control tasks. This included somatotopic mapping using light touch and deep pressure to his face region using one of the headache participants’ face masks that fortuitously happened to fit reasonably well. Informed consent was obtained for both participants following guidelines approved by the West Virginia University Institutional Review Board and in compliance with the Code of Ethics of the World Medical Association.

**Table 1:**
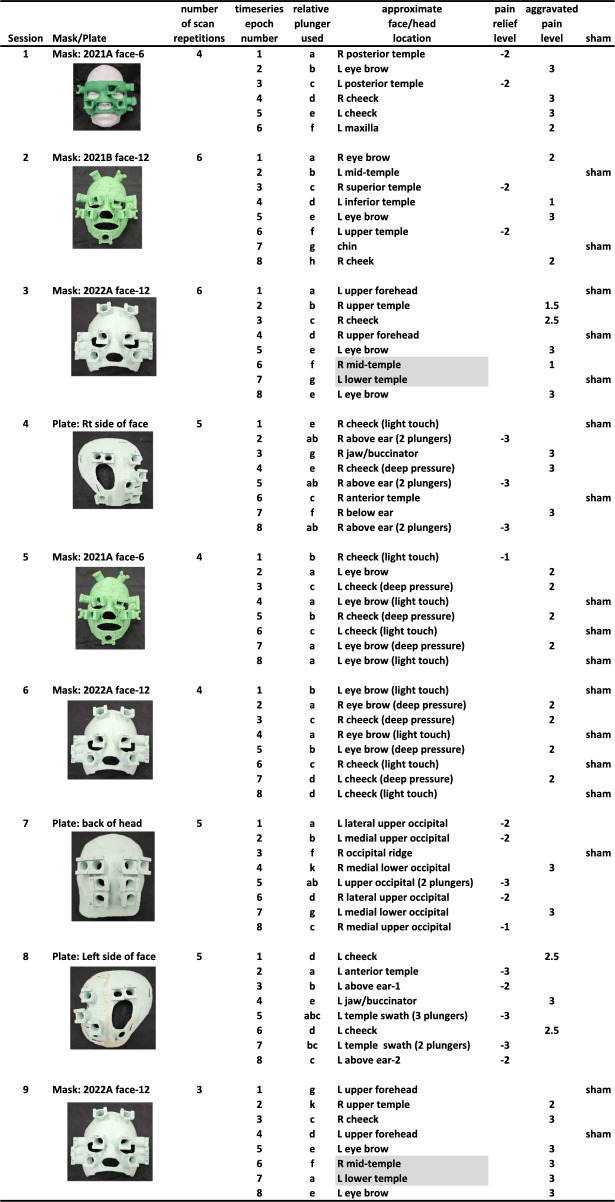
Listing of the nine fMRI sessions involving baseline pain aggravation epochs, including the mask/plates used, the approximate locations on the craniofacial locations stimulated, and the effect on pain modulations (relief, aggravation, or sham). In total, 18 epochs entailed partial relief, 35 epochs entailed aggravating baseline pain, and 17 epochs had no effect on modulating pain. Light gray cells highlight a minor discrepancy in the degree of pain rating across sessions that used the same face mask, likely due to precise fitting of the mask together with head placement and padding within the MRI headcoil

### 3D-printed MRI-compatible face/head masks

To systematically apply deep pressure to different locations on the headache participants’ face and head, we constructed several customized three-dimensional (3D) MRI-compatible masks [45]. A T1-weighted MRI of the NDPH headache participant was used to derive a 3D rendering of his head using 3D Slicer software (version 4.10.2, https://www.slicer.org/) [46], exporting a standard tessellation language (STL) file that was further edited (e.g. Figure 1B) using Meshmixer 3D visualization software (Autodesk Meshmixer, RRID:SCR_015736; http://www.meshmixer.com). With Meshmixer freeware, portions of the face, the left and right side of the face, and the back of the head region, were then expanded radially outward from the 3D head rendering and then computationally thickened to generate robust custom fitting face masks and skull plates. The masks/plates were computationally fitted with 6 to 12 inverted syringe plunger holders (Fig. 1C; “Pawlowski joints”) that could house a standard Luer-Lok syringe inverted as a plunger (Becton, Dickinson and Company, New Jersey, USA). The locations of the plungers were optimized to include a combination of craniofacial locations expected to elicit an increased, decreased, or neutral effect on the headache participant’s baseline chronic headache pain. The plungers also had to be placed at locations on the mask/plate that avoided contact with the rungs of the MRI headcoil(s) being used (e.g. Figure 1F). 3D masks/plates were exported into Cura software (Ultimaker Cura, version 4.10.0, accessed July 2021, available at: https://github.com/Ultimaker/Cura) to prepare gcodes for printing with a Creality Ender 5 Plus 3D printer with a 35 × 35 × 40 cm^3^ build volume. Masks were fitted with Velcro straps to help secure placement around the head within the MRI headcoil.

Up to eight plunger tube units were prepared (Fig. 1G), each consisting of two syringes (5, 6 or 10 ml) connected to T-intersection release valves, and each pair of syringe-valve ends were interconnected by ten 48-inch Luer-Lok polyvinyl chloride (PVC) tubes. This configuration allowed plunger tube units to extend from the MRI control room through a patch panel and into the MRI scanner bore isocenter around the participants’ head. Each plunger tube unit was filled with water colored with food dye. Different colors were used, together with a plexiglass housing, to facilitate calibration of each plunger unit and determine how many milliliters of fluid displacement were needed to produce sufficient pressure on the participant’s face/head to modulate his baseline pain. Air pockets were removed immediately prior to scanning to help reduce the lag times (~ 1–2 s) required for the hydraulic plunger to engage and disengage. A comparable degree of pressure was used at each plunger unit location, depressing the skin roughly orthogonal to the head, which was verified verbally by the participant (typically an increase in pressure rated at + 2 or + 3 out of 10). This amount of pressure typically required 1 to 4 ml of fluid displacement. Plunger displacement volumes that visibly moved the mask were avoided. Different plunger unit locations, and/or combinations of plungers, and/or light touch that did not modulate baseline pain, were implemented using the various masks/plates over the nine sessions (Table 1). For each plunger unit (of each mask/plate) a relative rating of whether the deep pressure partially relieved, aggravated, or had no effect on his baseline pain was recorded (Table 1; right-most columns). He reported his baseline pain modulation ratings on a 1 to 10 scale verbally during setup and calibration time immediately prior to each fMRI scanning session, and assessed again midway through or immediately after the scanning session to verify pain rating stability. He typically rated aggravation as a + 1, + 2, or + 3 increase from baseline chronic pain. Similarly, he rated partial relief to the same relative degree, typically as a −1, −2, or −3 decrease from baseline pain. If his midway or post-scanning assessment changed, then an average was entered (e.g., 2.5). His overall baseline chronic pain across visits varied from an overall rating of 1 to 4 (out of 10), and his overall baseline pain rating each day did not change after fMRI scanning after any of the neuroimaging sessions.

### MRI acquisition procedures

The imaging was conducted on two MRI scanners. All baseline pain modulation mapping sessions were conducted with a 3 Tesla Siemens Magnetom Prisma with a 28-channel radio frequency (RF) headcoil (20-channel head plus 8-channel neck), which was compatible with an electrostatic ear-bud system (STAX; Model S14, Sensimetrics Corp., Malden MA) that was used to communicate with a participant for plunger stimulation calibrations. Whole brain T1-weighted anatomical MR images were collected using a magnetization-prepared rapid acquired gradient-echo (MPRAGE) pulse sequence (1.5 mm sagittal slices, 0.625 × 0.625 mm^2^ in-plane resolution, TI = 1100 ms). A 3 Tesla Siemens Verio MRI scanner with 12-channel headcoil was used for a spoken language processing fMRI scan plus some additional equipment testing and calibration.

### Functional MRI brain mapping

Nine separate fMRI scanning sessions were conducted that used On/Off block paradigm scans with the hydraulic plunger systems (Table 1). Cycles of 20 s On-periods (epochs) flanked by 20 s Off-periods of rest with no plungers contacting the skin (typically 8 cycles with a duration of 5′46″ min each), and 3 to 6 repetitions of that scan. For a given epoch, different face and head regions were gently depressed by the back end of a 5 or 6 ml syringe plunger in a manner that would consistently (A) modulate (decrease or increase) his baseline chronic headache pain through deep pressure, (B) lightly touch the skin (including some of the same aggravation point locations) to a degree that was felt but did not modulate pain (“sham pressure-levels”), or (C) be felt as deep pressure while using similar levels of intensity (fluid volumes) as with the pain-modulating sites but in face/head locations that had no effect on his baseline chronic pain (“sham locations”). To minimize effects of pain-related anticipation or expectation [47], a randomized sequence of plunger locations [relief, pain, sham] was selected for each scanning session as depicted in Table 1 (columns 6–9).

### Spoken sentence processing

Earlier studies have suggested a possible link between language lateralization and headaches [48, 49]. Thus, we sought to identify brain regions related to language processing. As described previously by our group [50], we implemented a passive spoken phrase listening paradigm during a separate fMRI scanning session. Over three scans, 108 unique spoken phrases were presented (neutral tone by a female speaker; six phrases per 20 s On-period flanked by 20 s silent Off-periods). Stimuli were delivered using a Windows PC computer, with Presentation software (version 11.1, Neurobehavioral Systems Inc., Berkeley, CA) via a sound mixer and ear-buds (Model S14, Sensimetrics Corp., Malden MA). Stimulus loudness was set to a comfortable level. Eyes were closed during the scanning runs. This experiment revealed activation in brain regions consistent with a left-lateralization for semantic processing of spoken language (data not shown).

### Functional MRI Data analysis

All fMRI data were initially processed using Analysis of Functional NeuroImages (AFNI) and associated software plug-in packages (http://afni.nimh.nih.gov/) [51]. To address head motion issues (participant movement and possible head displacements caused during plunger applications), head translations and rotations were globally corrected using 3dvolreg plug-in software. Voxels were subjected to a Gaussian spatial blurring of 6 mm [52]. Modeling a 6 s hemodynamic delay, BOLD signals were then converted to percent signal change on a voxel-wise basis relative to rest events for each run. The scanning runs for each respective fMRI task condition were averaged. Multiple linear regression analyses were performed (3dDeconvolve software) to reveal voxels showing activation correlated with On-period epochs providing partial relief, aggravating baseline pain, or having no effect on baseline pain (sham). The headache participant provided relative ratings for degree of relief or pain aggravation (Table 1). For several session datasets the BOLD signal was modeled on the relative degree ratings versus a binary model (pain or relief), which produced no significant differences in outcomes. Thus, for simplicity, we opted to model BOLD signal responses using a binary waveform regardless of degree of relief or pain aggravation rating level. Functional and anatomical brain volumes were manually aligned to standardized Talairach space [53] using AFNI software for convenience of *t-test* analyses, but were also transformed to MNI152 Talairach space for CONN rsfMRI analyses.

To identify brain regions of interest in response to pain epochs or relief epochs, relative to sham condition epochs, a series of *t-tests* were performed. Each fMRI dataset had some degree of grid-like phase encoding/decoding spatial artifacts (also evident in field distortion maps) due to the introduction of water at some of the plunger locations near the head (not illustrated). To address this confound, each of the ROIs identified in the combined analysis that included all nine scanning sessions were further assessed visually to determine if they contained excessive local spatial artifacts that may have impacted that ROI; datasets where a clear local spatial artifact overlapping a given ROI was present were excluded and a subsequent *t-test* analysis for that ROI was then conducted. Each of these second-level analyses were then balanced as closely as possible across sessions for number of contrast control conditions, including sham location epochs, sham pressure-level (light touch) epochs located at aggravation points that did not affect baseline pain, and/or the relief epochs for two of the sessions otherwise without shams (Table 1, Sessions #1 and #8), all of which were measured relative to the baseline control of averaged BOLD signals from the flanking Off-periods when there was no contact to the skin by any of the plungers.

Different approaches were used to derive maps of somatotopically organized cortex in response to the face and head plunger pressure points that did not modulate baseline pain. One was to map the headache participant’s responses to the sham locations relative to baseline rest, revealing responses to deep pressure sensation of craniofacial locations in the absence of any pain modulation. A second was repeating an fMRI pain-mapping session (using identical parameters to session #3) except only using light touch at each of the plunger locations, wherein the plunger was felt on the skin but did not elicit pain or relief at any time during the scanning session. Finally, a healthy control participant was recruited who performed the identical series of both the deep pressure and a light touch fMRI session described above (of session #3) using one of the 3D-printed masks that happened to adequately fit his face.

### Resting state functional connectivity MRI (rsfMRI)

Functional connectivity during rest (with eyes closed) was assessed during five separate rsfMRI scanning sessions obtained from different visits over the course of 12 months. Blood oxygen-level dependent (BOLD) signals were collected continuously during a resting state paradigm (ep2d: TR = 2600 ms, TE = 30 ms, FOV = 240 mm), wherein whole-head brain volumes were collected (57 axial slices, 2.5 × 2.5 × 2.5 mm^3^ resolution), with 300 measurements per scanning session (13 min, 14 s duration). A contiguous 13 + min scan was conducted to capture potential low frequency fluctuations and increase intersession reliability [54].

The five brain regions revealed from the fMRI aggravated baseline pain mapping sessions (see Results) were used as primary ROIs for the below rsfMRI analyses. Guided by drawings from a neuroanatomy atlas [55], a bilateral periaqueductal gray (PAG) volume, plus left and right thalamus volumes, were derived from one of the headache participant’s T1 MPRAGE scanning datasets. After conducting multiple analysis variations with these ROIs, we opted to include the PAG anatomical ROI to facilitate interpretation given its presumed connectivity with the cerebellum [56] and given earlier studies implicating the PAG and cerebellum in chronic pain. An examination of subdivisions within the small volume PAG focus was beyond the scope of this study.

**Fig. 2:**
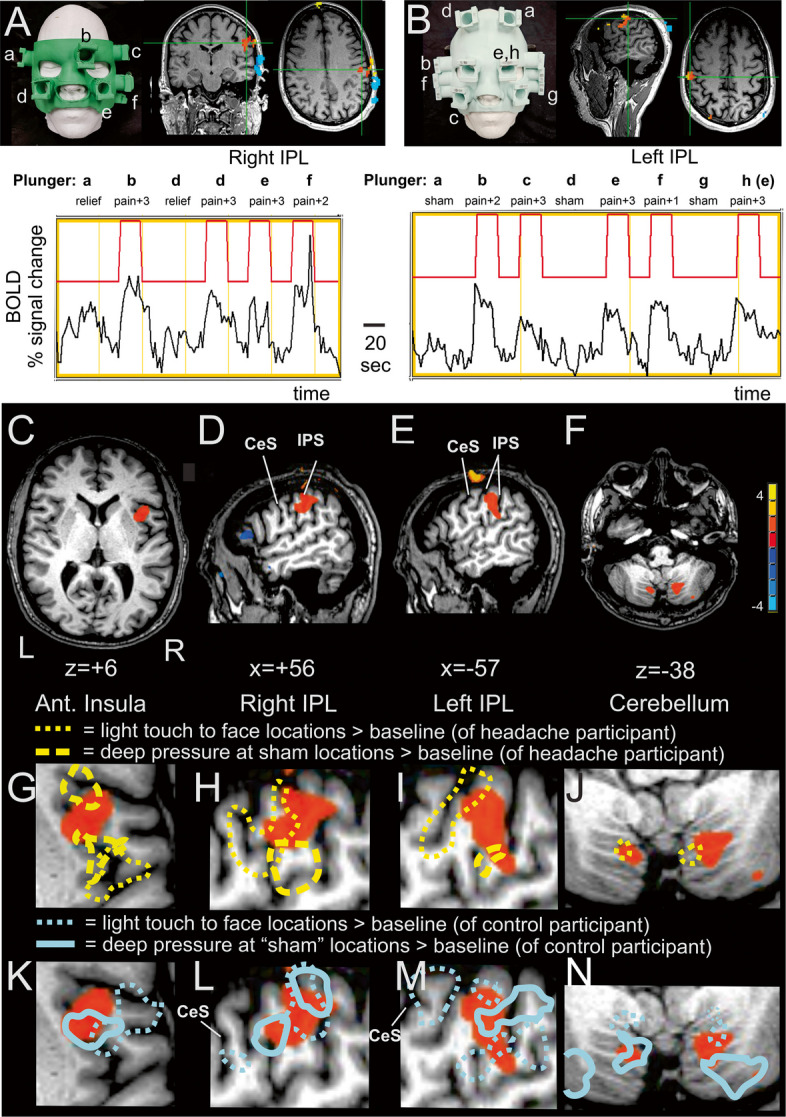
Brain regions showing significant increases in activation (BOLD signal) in response to transiently increasing (aggravating) the headache participant’s baseline chronic pain during application of deep pressure to various face and head locations (i.e., red areas from Fig. 1A) in contrast to sham (or relief) locations. Example timeseries data from two fMRI scanning sessions illustrating plunger sequences to the face and BOLD signal responses in (**A**) the right IPL (uncorrected *p* < 10^–13^, from session #1), and (**B**) the left IPL (uncorrected *p* < 10^–17^, from session #3). Signals on the skull exemplify some of the single session noise due to head motion and/or plunger displacement artifacts. Brain regions significantly activated after combining data across fMRI sessions, including the (**C**) right anterior insula, (**D**) right IPL, (**E**) left IPL, and (**F**) bilateral cerebellum. (**G-J**) Expanded views of the ROIs with overlays (yellow outlines) depicting somatotopic mapping from the headache participant’s light touch and deep pressure control conditions (see key). (**K-N**) Somatotopic mapping overlays from a healthy control participant in response to light touch at all plunger locations (cyan dotted outlines), and during deep pressure at the same “sham” locations as the headache participant (cyan solid outlines). CeS = central sulcus, IPS = intraparietal sulcus. Refer to text and Table 2 for other details

Two separate resting state analysis approaches were performed to assess the functional connectivity characteristics of the above defined “aggravated baseline pain” network. Addressed in turn below, the first analysis assessed instantaneous functional connectivity using a conventional approach (using CONN toolbox software), and the second assessed effective connectivity implementing a more exploratory approach (using 1dSVAR AFNI software), which assesses directionality of effect paths using Granger causality techniques. Because the headache participants’ overall baseline chronic pain was queried just prior to four of the rsfMRI sessions, this allowed for assessment of potential state-dependent differences between datasets acquired on two of the visits when he was experiencing a relatively greater degree of overall baseline chronic pain (rated at 3 out of 10) versus two visits when he reported a relatively lesser degree of chronic pain (rated at 1 or 2 out of 10).

### Functional connectivity analyses using CONN

Functional and structural images were analyzed using a MATLAB/SPM-based software CONN (version 22.a) [57–59]. Data were preprocessed using a standard pipeline, described in the subsections below, using Statistical Parametric Mapping software (SPM12, Wellcome Department of Cognitive Neurology, University College London) [60] running under MATLAB Release 2024a (The MathWorks, Inc., Natick, MA, United States). We transformed the five fMRI-derived ROIs plus the anatomically-derived PAG ROI to the MNI152 brain atlas as the template for this analysis.

Raw MRI anatomical images and rsfMRI functional runs were visually inspected across the five scanning sessions (that had full brain coverage, including the cerebellum) using the CONN toolbox for abnormalities or missing data. Anatomical data were segmented into grey matter, white matter, and CSF tissue classes using SPM unified segmentation and normalization algorithm [61] with the default IXI-549 tissue probability map template.

#### Denoising

Functional data were denoised using a standard denoising pipeline [62] including the regression of potential confounding effects characterized by white matter timeseries (5 CompCor noise components), CSF timeseries (5 CompCor noise components), session effects and their first order derivatives (2 factors), and linear trends (4 factors) within each functional run, followed by bandpass frequency filtering of the BOLD timeseries between 0.008 Hz and 0.09 Hz. CompCornoise components within white matter and CSF were estimated by computing the average BOLD signal as well as the largest principal components orthogonal to the BOLD average within eroded segmentation masks [63, 64]. From the number of noise terms included in this denoising strategy, the effective degrees of freedom of the BOLD signal after denoising were estimated to range from 122 to 122 (average 122) across all subjects (Nieto-Castanon, 2020).

#### First-level analyses

ROI-to-ROI connectivity matrices (RRC) were estimated characterizing the patterns of functional connectivity with the five ROIs related to the aggravated pain network. To compensate for possible transient magnetization effects at the beginning of each series of scans, individual scans were weighted by a step function convolved with an SPM canonical hemodynamic response function and rectified.

#### Group-level analyses

The Second-level analyses (Group level; wherein all rsfMRI datasets were from the headache participant across different months), applied to each of the above first-level analyses, were performed using a General Linear Model (GLM) [62]. For each individual voxel a separate GLM was estimated, with first-level connectivity measures at this voxel as dependent variables (one independent sample per session), and intersession-level identifiers as independent variables. Voxel-level hypotheses were evaluated using multivariate parametric statistics with random-effects across sessions and sample covariance estimation across multiple measurements. Inferences were performed at the level of individual clusters (groups of contiguous voxels). Cluster-level inferences were based on parametric statistics from Gaussian Random Field theory [62, 65]. Primary results were derived by thresholding using a combination of a cluster-forming *p* < 0.05 voxel-level threshold plus a false discovery rate error corrected p-FDR < 0.05 cluster-size threshold [66]. Cluster-level inferences based on Threshold Free Cluster Enhancement (TFCE) analyses [67] aim at removing the dependency of other cluster-level inference methodologies on the choice of an a priori cluster-forming height threshold.

### Effective connectivity analyses using structural vector autoregression (SVAR)

The causal influences that the five fMRI-derived ROIs plus anatomically-derived PAG ROI may have on one another (effective connectivity) were analyzed using structural vector autoregression (SVAR; AFNI program 1dSVAR.R) methods [37, 38, 50, 68–70]. This approach, based on Granger causality analysis, models both instantaneous and lagged effects using a unified analytical framework, and we focused on examining lagged effects (1 lag = 1 TR = 2.6 s) among the same brain regions described above (using CONN) with no a priori assumption about properties of the ROIs apart from their involvement in pain processing. Statistical analysis was implemented using R software [71]. Residual time series for each rsfMRI session for the selected ROIs were exported and used as input time series for the network modeling. For the SVAR approach, signals were processed without the traditional ~ 0.008–0.09 Hz bandpass filtering [62, 72] to avoid potential biasing confounds [73]. Statistical analyses were performed at the individual subject level, and ‘group’ effects (combined datasets of the one headache participant) were analyzed through linear mixed-effects meta-analysis using path coefficients for each session. This analysis provided estimates of an average path coefficient (α) and their respective *t*-values of each session in the group, and its statistical significance (p-value, two-tailed, uncorrected) for each interaction within the network. Multiple comparisons were corrected by Bonferroni procedure, multiplying the uncorrected p-value by the number of possible directional pairs of the six ROIs (30 total).

## Results

The main finding of the present study was that modulating the baseline pain of a chronic headache patient using MR-compatible plunger systems can reveal brain network activations associated with changes in the perception of neuropathic pain. Regarding our first hypothesis, no brain regions survived statistical significance for revealing differential activation in response to deep pressure that provided partial relief of baseline pain (data not shown). This null result was likely due, at least in part, to the need to compress larger surface areas of tissue along the sides of the head, and/or with greater pressures, for our headache participant to achieve a significant degree of relief while lying in the scanner, and thus relatively greater degrees of spatial artifacts were present during these epochs (see Limitations and Future Directions).

**Fig. 3:**
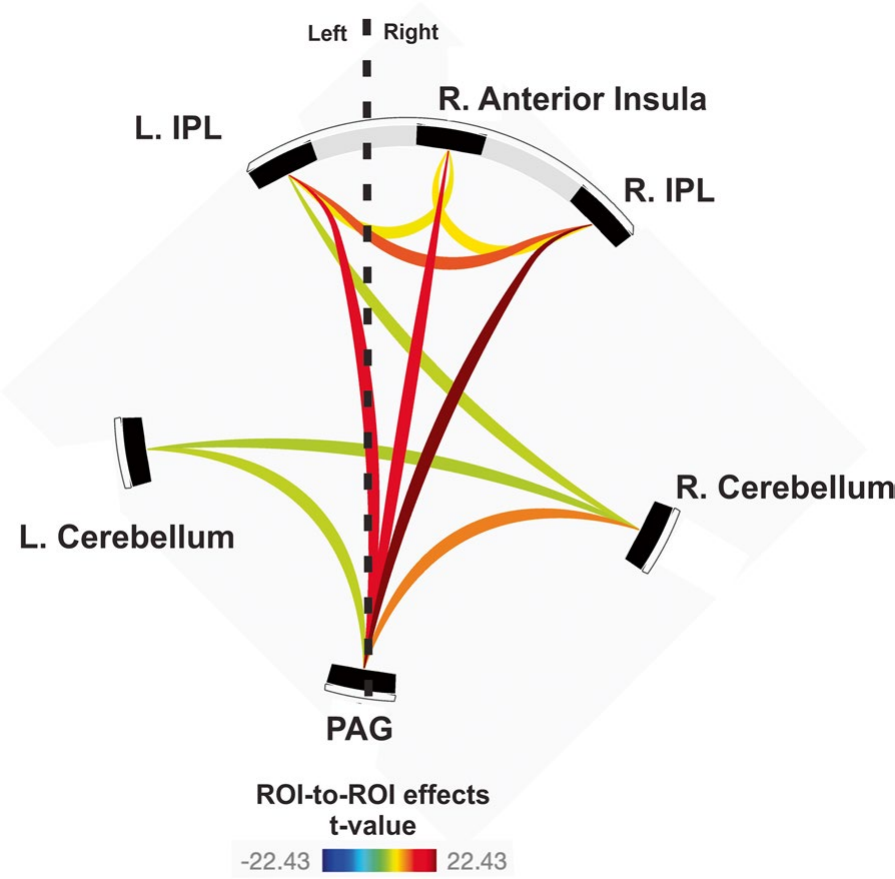
Mean rsfMRI functional connectivity network of the aggravated baseline pain network (ROIs from Fig. 2C-F) plus an anatomically defined periaqueductal gray (PAG) region of interest. All data at *p* < 0.05 voxel threshold (two-sided), and *p* < 0.05 FDR-corrected. Refer to Table 3 and text for other details

**Fig. 4:**
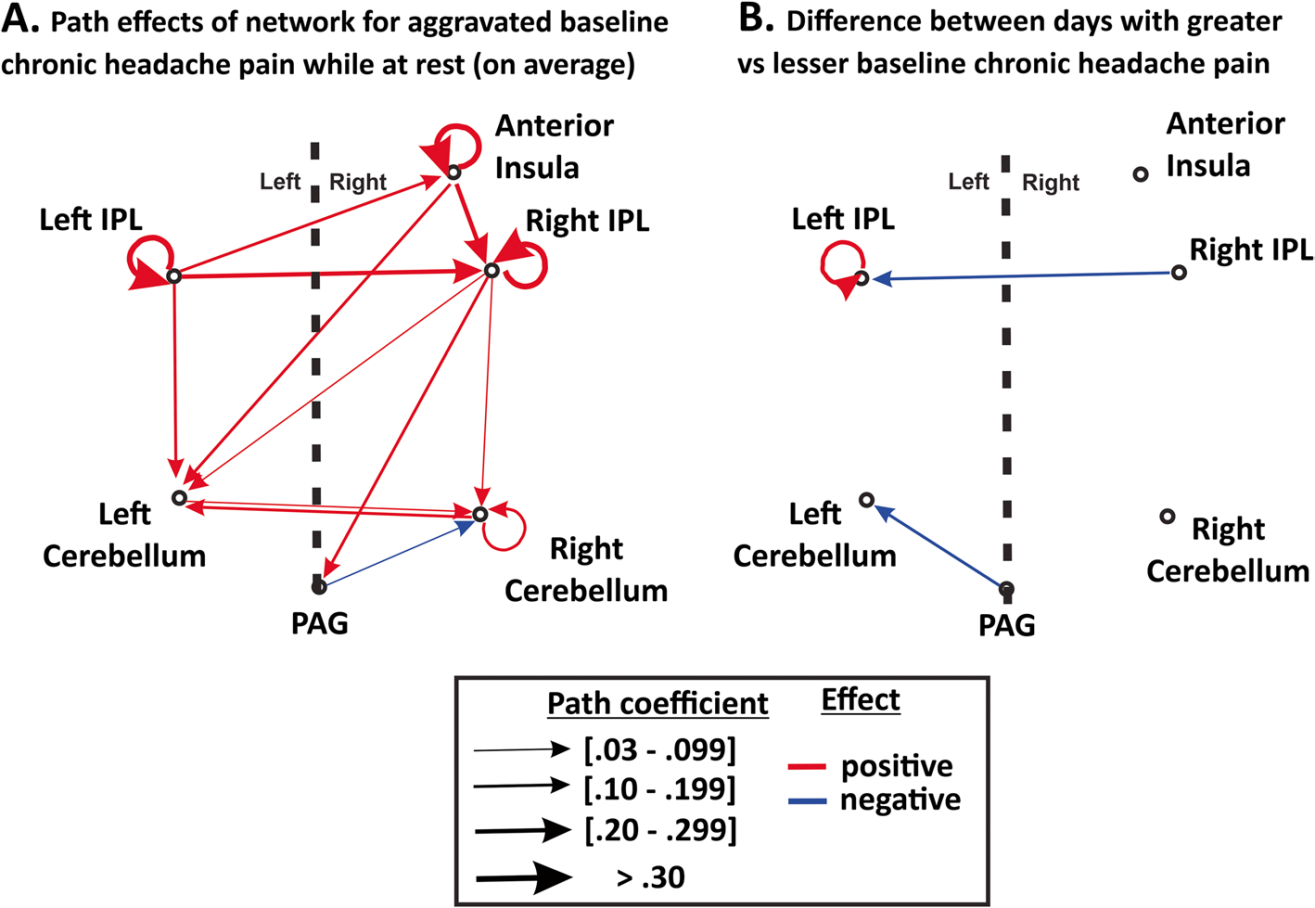
Results of the multivariate first-order structural vector autoregression (SVAR) analysis of rsfMRI data amongst the aggravated baseline pain network (ROIs from Fig. 2C-F) plus the periaqueductal gray (PAG) ROI. (**A**) Results summarizing meta-analytic one-group statistics for path coefficients (α > 1) when combining all five separate rsfMRI scans collected across separate months. Arrow thickness represents path coefficient strength (see Table 4 for *p*-values). (**B)** Using the same ROIs, a two-group analysis was conducted using 4 of the 5 rsfMRI scans, revealing differences among path coefficients on days the headache participant had relatively greater degrees of overall baseline chronic pain (see Table 5 for *p*-values). Refer to text for other details

Regarding our second hypothesis, however, five brain regions showed significant activation in response to deep pressure at face and head locations that aggravated the headache participant’s baseline chronic pain relative to sham conditions as critical controls (Fig. 2, Table 2). The five ROIs were further assessed for functional roles using rsfMRI datasets as independent measures of both the functional connectivity (Fig. 3, Table 3) and effective connectivity (Fig. 4, Tables 4 and 5) within this network, which further revealed state-dependent differences on days the headache participant had relatively greater degrees of baseline chronic headache pain.

**Table 2:** Group activation centroids in Talairach (AFNI) coordinate space for *t-test* cortical ROI foci from collective fMRI datasets (corresponding with Fig. 2C-F), depicting brain regions significantly activated when aggravating baseline chronic headache pain relative to sham conditions. See text for other details

	Talairach coordinates			Volume
Anatomical location	x	y	z	(mm^3^)
Right Anterior Insula	40	1	11	3162
Right Intraparietal lobule	57	−28	35	2179
Right Cerebellum	18	−64	−39	1392
Left Intraparietal lobule	−60	−27	29	1195
Left Cerebellum	−10	−67	−38	317

**Table 3:** Statistical cluster analyses from CONN functional connectivity analyses of network changes of the ROI-to-ROI functional connections of the aggravated baseline pain network plus periaqueductal gray (PAG). Each entry corresponds to a given curve in the connectome ring of Fig. 3

Aggravated pain network + PAG		Statistic	p-FDR	p-FWE
**Cluster 1**		F(1,4) = 412.57	0.00004	0.00024
PAG	Right. IPL	T(4) = 22.43	0.00002	0.00012
PAG	Left IPL	T(4) = 17.87	0.00006	0.00014
PAG	Right Anterior Insula	T(4) = 15.97	0.00009	0.00015
**Cluster 2**		F(1,4) = 111.87	0.00045	0.00122
PAG	Right Cerebellum	T(4) = 10.58	0.00045	0.00057
**Cluster 3**		F(1,4) = 103.67	0.00052	0.00122
Right IPL	Left IPL	T(4) = 12.14	0.00228	0.00380
Right IPL	Right Anterior Insula	T(4) = 6.18	0.00348	0.00580
**Cluster 4**		F(1,4) = 16.60	0.01517	0.02655
PAG	Left Cerebellum	T(4) = 4.07	0.01517	0.01517
**Cluster 5**		F(1,4) = 12.66	0.02360	0.03308
Right Cerebellum	Left Cerebellum	T(4) = 3.56	0.02360	0.03938
**Cluster 6**		F(1,4) = 10.10	0.03362	0.03922
Right Cerebellum	Left IPL	T(4) = 3.96	0.01665	0.03938

**Table 4:** Mean path coefficients and significance level for each connection from SVAR effective connectivity analysis of the aggravated baseline pain network plus PAG upon grouping five separate rsfMRI scanning session datasets. P-values and Bonferroni corrected *P*-values at α = 0.05 for 15-pairwise corrections are indicated. Refer to text and corresponding Fig. 4A for other details

Connection		Mean path coefficient	*P*-value	*P*-value
**From**	**To**			**corrected**
Right Ant. Insula	self-loop	0.336	0.0000	< 0.001
Right Ant. Insula	Right IPL	0.227	0.0000	< 0.001
Right Ant. Insula	Left Cerebellum	0.168	0.0388	
Right IPL	self-loop	0.309	0.0000	< 0.001
Right IPL	Right Cerebellum	0.084	0.0006	
Right IPL	PAG	0.176	0.0348	
Right IPL	Left Cerebellum	0.097	0.0065	
Left IPL	Right Ant. Insula	0.123	0.0000	< 0.001
Left IPL	Right IPL	0.260	0.0000	< 0.001
Left IPL	Left Cerebellum	0.116	0.0031	
Left IPL	self-loop	0.519	0.0000	< 0.001
Right Cerebellum	self-loop	0.199	0.0000	< 0.001
Right Cerebellum	Left Cerebellum	0.184	0.0000	< 0.001
Left Cerebellum	Right Cerebellum	0.074	0.0082	
PAG	Right Cerebellum	−0.030	0.0121

**Table 5:** Path coefficients and significance level for each connection from SVAR effective connectivity analysis comparing two separate rsfMRI sessions occurring on days in which the participant reported a relatively greater degree of baseline chronic headache pain. P-values and Bonferroni corrected *P*-values at α = 0.05 for 15-pairwise corrections are indicated. Refer to text and corresponding Fig. 4B for other details

Connection		Mean path coefficient	*P*- value	*P*- value
				corrected
From	To			
PAG	Left Cerebellum	−0.120	0.0008	< 0.023
Right IPL	Left IPL	−0.250	0.0460	
Left IPL	Self-loop	0.186	0.0450	

### Functional MRI mapping of baseline pain aggravation

Examples of raw data from two of the nine fMRI sessions are illustrated, including BOLD signal responses to pain versus relief epochs in the right inferior parietal lobule (IPL, uncorrected *p* < 10^–13^; Fig. 2A; Table 1, session #1), and responses to pain versus sham locations in a voxel in the left IPL (uncorrected *p* < 10^–17^; Fig. 2B; Table 1, session #3). Upon inspection, these (and all subsequent) fMRI scanning datasets contained at least some degree of spatial grid-like artifact signals that were clearly related to changes in volume of water in some of the hydraulic plunger locations; this appeared to be due to linear k-space phase encoding/decoding and typically affected BOLD signal in a subset of parasagittal planes (not illustrated). Thus, our approach had become that of data triangulation, collecting datasets across multiple fMRI sessions using different face masks and skull plates, and/or different face mask plunger configurations (Table 1). We then combined fMRI datasets using *t-tests* across nine fMRI sessions to isolate to the extent possible the effects of pain modulation (relief or aggravation) from the effects of (i) general deep pressure somatotopic stimulation (responses to non-aggravating deep pressure at face/head locations that did not modulate pain), (ii) light touch to pain aggravation points (at pressure levels that did not modulate pain), and (iii) spatial artifacts associated with the specific configurations of different mask/plate plunger devices. After candidate ROIs were identified by *t-test* as being responsive to the aggravated pain conditions relative to Off-period baseline rest across all nine fMRI sessions (two-tailed, uncorrected *p* < 0.05), those sessions that contained grid-like or excessive spatial displacement artifacts that overlapped a candidate ROI were excluded and the data was reassessed by *t-tests* using the remaining viable sessions for that ROI.

Five brain regions consistently showed significantly greater activation (BOLD signal change) in response to deep pressure at locations that aggravated baseline chronic pain relative to sham condition epochs. The goal was to balance as closely as possible the number of sessions that included pain-modulating epochs to the number of sessions including sham condition epochs across the two-sample *t-test* analyses, thereby assuming homoscedasticity, and applying a minimum cluster size threshold to help correct for multiple comparisons. One significantly activated region was the right anterior insula (Fig. 2C), which was activated in six of the nine sessions (sessions #1, 5, 6, 7, 8, and 9) involving aggravated pain epochs relative to four sessions (#4, 5, 6, and 7) with sham conditions as critical controls (two-tailed *t-test*, uncorrected *p* < 0.001, with a minimum cluster size of 180 mm^3^). Second was a right intraparietal lobule (IPL) focus (Fig. 2D), activated in a different set of six of the nine aggravated pain sessions (#1, 2, 5, 7, 8, and 9) relative to six sessions (#1, 2, 4, 5, 7, and 8; with session #1 and #8 using the relief epochs) as sham conditions (two-tailed *t-test* uncorrected *p* < 0.01, with a minimum cluster size of 1900 mm^3^). Third was a left IPL focus (Fig. 2E) in seven of the nine sessions (#1, 2, 3, 4, 5, 6, and 9) involving aggravated pain epochs versus seven sessions (#2, 3, 4, 5, 6, 7, and 9) with sham condition epochs (two-tailed *t-test* uncorrected *p* < 0.04, with a minimum cluster size of 900 mm^3^). Finally, there were left and right cerebellar foci (Fig. 2F; overlapping lobule VIIIb) in all nine sessions relative to seven sessions (#2, 3, 4, 5, 6, 7, and 9) with sham control conditions (two-tailed *t-test* uncorrected *p* < 0.05, with a minimum cluster size of 300 mm^3^). The coordinates of these five consistently surviving ROIs are indicated in Table 2 and are herein referred to as the “aggravated baseline pain” network for this headache participant.

To provide further context regarding the functional locations of the above five ROIs, several somatotopic mapping control scanning conditions were conducted. One control condition identified cortical regions of the headache participant that were responsive only to deep pressure at sham locations of the face/head relative to baseline rest using the same session artifact exclusion criteria as described above. The ‘sham locations’ on the face and head that did not modulate baseline pain were necessarily in different locations from ‘aggravation points’. Thus, brain activation in response to the sensation of aggravated baseline pain could not be dissociated from the sensation of deep pressure at those same locations. Notwithstanding, analyses of brain activation in response to deep pressure at sham locations revealed activation foci (from sessions #4, 5, 6, and 7, two-tailed *t-test* uncorrected *p* < 0.05) that juxtaposed and partially overlapped the three cortical foci related to baseline pain aggravation (Fig. 2G-I; dashed yellow outlines overlapping orange-red foci). Interestingly, the cerebellar aggravation baseline pain foci and nearby cerebellar territories did not show significant activation to deep pressure at sham locations (from any combination of sessions versus baseline rest), even at much lowered thresholds (Fig. 2J), further distinguishing the cerebellum’s possible differential functional role(s) in this network.

Another control condition entailed having the headache participant undergo a separate ‘light touch only’ fMRI session that used the identical face mask and plunger sequence from one of the earlier deep pressure fMRI sessions (Table 1; repeat of session #3 with only light touch epochs). The light touches by the plungers contacting the skin did not affect his baseline chronic pain at any of the locations. Non-aggravating light touch to the face from all plunger locations, relative to baseline rest, revealed activation foci along the post-central sulci and gyri (Fig. 2G-J, dotted yellow outlines, uncorrected *p* < 0.05), consistent with typical somatotopy of the face region [74]. In the left and right IPL regions light touch activated cortices in the post-central sulcus/gyrus regions (Fig. 2H-I; dotted yellow outlines), while deep pressure stimulation to sham locations activated regions located further posteriorly in parietal cortices (cf. dotted versus dashed yellow outlines). The cerebellar foci for baseline pain aggravation did, however, show partial overlap with the light touch to the face locations (Fig. 2J) despite their not being activated by the sham deep pressure location condition described earlier. Overall, the light touch versus deep pressure stimulation mapping sessions revealed different patterns of activity within the somatosensory cortices (left and right IPL) plus right insula and cerebellar foci.

An additional set of control conditions entailed recruiting a healthy control participant who happened to fit in the 3D-printed face mask used in session #3, and having him conduct both the ‘deep pressure’ and ‘light touch only’ scan sessions using the identical scanning parameters and epoch sequences as described above for the headache participant. Both participant’s respective datasets were converted to a common Talairach space (in AFNI) to facilitate comparisons. For the deep pressure stimulation fMRI session, similar degrees of deep pressure intensity (based on verbal feedback and similar volumes of water displacement) were used as for the headache participant. For direct comparison, we mapped activation in response to the same ‘sham’ plunger locations as the headache participant relative to baseline rest (Fig. 2K-N, solid cyan outlines, uncorrected *p* < 0.01). This control participant’s activated foci also revealed some degree of partial overlap with the headache participant’s aggravated baseline pain network (solid cyan outlines overlapping orange-red ROIs), and with the headache participant’s deep pressure ‘sham locations’ activation (cf. Figure 2G-N, dashed yellow vs solid cyan outlines). Similarly, the ‘light touch only’ fMRI session with the control participant revealed somatotopic activation foci in the cortical and cerebellar regions (Fig. 2K-N, dotted cyan outlines; uncorrected *p* < 0.05) partially overlapping those of the headache participant (cf. Figure 2G-N; yellow versus cyan dotted outlines).

Overall, both the headache participant and control participant revealed somatotopic activation in regions near or partially overlapping the five ROIs activated during aggravation of baseline headache pain. Thus, the aggravated baseline pain regions (Fig. 2) were in locations consistent with representing, at least in part, primary (first-order) somatotopic representations of the face region, but also including some juxtaposed cortical and cerebellar regions that may reflect other somatotopic-related processing (see Discussion).

### Resting state functional connectivity of aggravated baseline pain regions

For the rsfMRI data, two methods were implemented to assess both the functional and effective connectivity amongst the five “aggravated baseline pain” network ROIs defined using fMRI (Fig. 2C-F). A bilateral periaqueductal gray focus (based on volumetric outline from one of the headache participant’s neuroanatomical scans) was additionally included in the presented analyses due to its known involvement in pain modulation circuits [40, 75]. The first rsfMRI analysis approach examined conventional instantaneous connectivity (using CONN), while the second approach entailed more exploratory analyses (using SVAR) to reveal effective connectivity and possible directional effects (information flow) as a circuit.

The ROI-to-ROI functional connectivity analysis using a spatial pairwise clustering method (using CONN software) revealed significant connections between the five aggravated pain network ROIs and the PAG (Fig. 3; two-sided voxel-wise *p* < 0.05, FDR-corrected at *p* < 0.05). This analysis notably revealed clusters involving the PAG, which showed connectivity to all five of the pain network ROIs, plus a functional cluster of cortical regions (Table 3).

To identify effective connectivity between the above six ROIs, we used a structural vector autoregression (SVAR) technique. After accounting for both contemporaneous and lagged effects the SVAR analysis revealed a set of effect paths when examining all five rsfMRI datasets (from separate visits) as a group (Fig. 4A, Table 4). A Bonferroni correction for multiple comparisons was conducted, with a new α < 0.05 for 15 pairwise comparisons as indicated in the Tables. During rest the left IPL exhibited positive effect paths (effective connectivity) to the right anterior insula, and both regions had positive effect paths to the right IPL as a terminal cortical focus. These three cortical ROIs also exhibited strong positive self-loop effect paths. The cortical network had positive effect paths to the non-cortical network (bilateral cerebellum and PAG), but not vice versa. The left and right cerebellar foci had positive effect paths to one another, and the PAG had a negative effect path to the right cerebellum. Overall, the SVAR-derived effective connectivity interactions amongst the six ROIs were consistent with the above analysis using (CONN) functional connectivity (cf. Figures 3 and 4A), but further revealed a possible circuitry of information flow.

Because our headache participant had different degrees of baseline chronic pain on different visits (months apart), we further tested for resting state network differences from two visits (sessions) when the headache participant’s pain was self-reported to be relatively greater (rated at a 3 out of 10 overall pain) versus two visits when his overall baseline pain was self-reported to be more mild (rated a 1 or 2 out of 10). Results from this intersession SVAR analysis (Fig. 4B, Table 5) revealed greater overall baseline chronic headache pain as being associated with a positive self-loop effect path within the left IPL, a negative effect path from the right IPL to the left IPL, and a negative effect path from the PAG to the left cerebellum.

## Discussion

In the present study, we mapped functional brain regions in response to transient modulations in baseline chronic pain sensations in a headache patient-participant with a long history of chronic pain associated with New Daily Persistent Headache (NDPH). There were two main findings. First, using novel MRI-compatible 3D-printed deep pressure delivery devices, stimulation to various face and head regions that aggravated his baseline pain (relative to sham conditions) led to significant activation in five brain regions, including the right anterior insula, left and right IPL, and bilateral ventral cerebellar cortices (lobule VIIIb), constituting an “aggravated baseline pain” network related to somatosensory processing. Conversely, no brain regions were observed in response to deep pressure at locations leading to partial relief from baseline pain, though this was at least in part due to technical limitations (see Limitations and Future Directions). Second, the above fMRI-defined pain-related network exhibited effective connectivity (information flow) as a potential circuit, including positive effect paths from the left IPL to the right anterior insula, and both regions had positive effect paths to the right IPL as an end-stage cortical ROI. This tripartite cortical network in turn was interconnected with the bilateral cerebellar and PAG regions, and only the PAG showed a negative effect path (to the right cerebellum) in this circuit. This circuit had additional psychophysiological characteristics in that on days the headache participant had relatively greater overall baseline chronic pain there was a significant positive self-loop effect path within the left IPL, a negative effect path from the PAG to the left cerebellum focus, and from the right IPL to the left IPL.

### Aggravated pain network

Collectively, the five ROIs that constituted an aggravated baseline pain network, as revealed through deep pressure stimulation to specific craniofacial locations, appeared to all be associated with a mix of first-order somatosensory processing stages, plus some possibly higher-order processing stages that were juxtaposed to, and contiguous with, those primary somatosensory-related regions. The sensation of deep pressure versus baseline pain modulation at the ‘aggravation points’ could not be dissociated by the headache participant. Nonetheless, deep pressure applied to the face in sham locations, for both the headache participant and corresponding locations for the healthy control participant, led only to partial overlap of activation in most of the five pain-related ROIs at the selected threshold settings (Fig. 2K-N, solid cyan and dashed yellow outlines). Interestingly, the cerebellar foci were not significantly activated by deep pressure to sham locations by the headache participant (Fig. 2J), ostensibly implicating these foci with additional non-typical sensory processing characteristics. Lobule VIIIb of the cerebellum, where the aggravated baseline pain foci were localized, has been implicated in secondary sensory processing in non-human primates [76, 77]. However, the fractured somatotopic nature of the maps in the cerebellum makes this comparison to group-level fMRI studies with humans more difficult to interpret [78, 79], and thus awaits further study.

In the headache participant, light touch at the aggravation points, plus other craniofacial locations, led to brain activation showing only a slight degree of partial overlap in the bilateral parietal and cerebellar foci, and no overlap in the right anterior insula (Fig. 2G-N, dotted outlines overlapping orange-red ROIs). In both the headache participant and control participant the light touch control session paradigms revealed activation foci that also partially overlapped the pain-related bilateral IPL and bilateral cerebellar foci (Fig. 2 H-J and L-N; dotted outlines overlapping red–orange foci). The light touch versus deep pressure activated regions located in the IPL and post-central gyri were consistent in locations reported as somatotopic maps for light touch versus deep pressure along primary somatosensory cortices [Brodmann Areas 3b, 3a, 1 and 2] [80–84]. The light touch versus aggravated baseline pain foci, however, were juxtaposed to one another in the right anterior insula at the selected threshold settings (Fig. 2 G, K). Overall, while distinguishing first-order from higher-order somatosensory processing was not possible with these data the differences in response profiles of the regions comprising the aggravated baseline pain circuit are suggestive of differences in the roles each may have in mediating the neuropathic perception of chronic NDPH pain.

Individually, the five aggravated baseline pain ROIs have previously been at least loosely implicated in chronic pain processing. In brief, the right anterior insula is widely reported to respond to the emotional awareness, attention, and salience detection of pain [2, 85], and similar to the present study has been reported to correlate with chronic back pain intensity when noxious thermal stimulation that aggravated the lower back pain was applied [17]. The left and right IPL foci (including the intraparietal sulcus, IPS) were similar in location to regions reported to show altered resting state (rsfMRI) activity in patients with persistent somatoform pain disorder (PSPD) relative to healthy controls [86], and to patients with chronic back pain during an attention demanding task [34].

The cerebellum has frequently been implicated in brain networks mediating, or affected by, chronic pain [8, 87–89], including PSPD [86], chronic neuropathic pain of the face [90], migraine [91, 92] and with NDPH [36]. Lobule VIII of the cerebellum, more specifically, was reported to show decreased coupling with the anterior cingulate cortex in pediatric patients with post-traumatic headache [93]. Overall, the present study not only contributes to this growing inventory of brain regions implicated in chronic pain processing, but further characterizes a potential circuit model.

The effective connectivity analyses (using SVAR) revealed a brain circuit related to baseline chronic pain processing (without aggravation) that may reflect the functional role(s) that each of these brain regions and/or their interconnections may have in mediating chronic pain perception. Though still exploratory, the SVAR signed-path coefficients generate estimates of the probable excitatory or inhibitory effects of the directed physiological influence [35, 94]. The independent resting state (rsfMRI) analyses with the five fMRI-derived aggravated pain network ROIs, plus periaqueductal gray (PAG) region, indicated that the left and right cerebellar foci received positive path effects (presumably, though not necessarily, excitatory) from the cortical network regions, and received negative path effects (presumably, though not necessarily, inhibitory) from the PAG (Fig. 4), suggestive of an imbalance of this circuit with particular regard to cerebellar function. The path effects of the left IPL were also distinctive in that this brain region was positioned as an inciter (“ring leader”) emanating path effects to the other cortical regions and ipsilateral cerebellum, yet receiving negative path effects (from the right IPL) on days the headache participant had relatively greater overall chronic headache pain.

So, what are the functional roles of the specific ROIs comprising this “aggravated baseline pain” network for this headache participant? One possibility is that the network of brain regions revealed may simply not be fundamentally related to his underlying chronic pain perception at all, in that the differential activation we revealed may only reflect some form of *somatic attention* to the change in the headache participant’s chronic pain when aggravated. At the other extreme, this network may be reflecting most of his neuropathological pain matrix, which is a network that is somehow caught up in a perpetual feedforward loop that subserves the chronic perception of neuropathic pain, and we were simply further aggravating it. Alternatively, the functional role(s) of some of these ROIs may lie somewhere between these two extremes. Notwithstanding, the brain regions comprising this headache participant’s aggravated baseline chronic pain network, which has presumably been a stable neuropathic network for over a decade since the sudden onset of his NDPH, now at least serve as candidate ROI sites for targeting with neuromodulation techniques. For instance, one may be able to mitigate pain by modulating the functioning of specific brain regions, recalibrating the circuit dynamics to ultimately abort or at least help disrupt the cycle that is giving rise to the chronic perception of pain.

### Limitations and future directions

Brain regions associated with transient and immediate partial relief from our headache participant’s chronic pain were not observed at a level of statistical significance when analyzed using session-level *t-tests*. This relative lack of finding was at least in part due to technical limitations with accessing the more difficult-to-reach craniofacial relief points along the sides of the head in the MRI headcoil (e.g. Figure 1A, green along sides and back of his head). Additionally, application of relatively greater surface areas and/or pressures were needed to truly attain a relief status, but this led to relief epochs that exhibited greater displacement artifacts and/or grid-artifacts (e.g., Table 1, note attempts at using multiple plungers simultaneously in sessions #4, 7, and 8). The ‘grid artifacts’ appeared to reflect linear k-space susceptibility signals, which were inevitably introduced to some degree when water was plunged into the field-of-view in the MRI headcoil, which was a technical hurdle that was difficult to overcome except by triangulating the outcomes across multiple fMRI sessions that used different masks/plates and/or plunger configurations.

The use of air in the tubes (a pneumatic system) was attempted, but the temporal lag for plunger movement and limited degree of deep pressure intensity that could be applied rendered this approach less effective with our apparatuses. The use of different liquids (e.g., mineral oil) and other pressure delivery systems (e.g., adjustable head bands, manual deep pressure stimulation) proved to be cumbersome in the MRI environment, though in principle more advanced equipment and/or use of other liquids (with more suitable permittivity and dielectric values) could mitigate some of the imaging artifacts we encountered. An additional option might be to use other k-space sampling techniques (e.g., spiral pulse sequence sampling) that could alter the degree and/or location of susceptibility artifacts introduced by equipment touching the face/head of a participant. Nonetheless, future studies with this or similar deep pressure delivery systems, together with headache participants’ who’s baseline pain can be readily, and transiently, modulated through somatosensory stimulation, would be warranted both to replicate our findings and to identify a potential ‘relief matrix’ mediating, or associated with, alleviation of chronic headache pain.

As with many case studies, another limitation was the lack of replication and limited neurotypical control measures. Given the technical difficulties of mapping deep pressure to craniofacial regions while lying in an MRI scanner, there is a relative lack of normative neuroimaging data regarding detailed face/head somatotopy to touch and to deep pressure. We were fortunate to have a control participant (though not age matched) who happened to fit well enough in one of the headache participants’ masks. Future headache mapping studies, and studies of chronic pain conditions that are modulated by deep pressure massaging to the head and face, would likely benefit from finer-grained normative fMRI mapping of craniofacial somatosensory responses to light touch versus deep pressure.

## Conclusion

In conclusion, results from the present study supported the hypothesis that transiently modulating the baseline chronic pain of a headache participant can yield significant changes in brain activation that are detectable using fMRI. Our approach revealed an aggravated baseline pain network of regions that appeared to involve primary plus potentially higher-order somatosensory-related processing. For the first time, this headache participant (and his health care team) could observe objective measures and psychophysiological characteristics underlying his chronic headache pain—a potential biomarker for his otherwise invisible NDPH headache disorder. Moreover, rsfMRI outcomes further enabled the identification of different effect paths (flow of information) amongst the pain processing brain regions of interest, which may constitute a basic circuit model that can be systematically tested for function or pathology using neuromodulation techniques.

## Data Availability

The data that support the findings of this study are available from the corresponding author upon reasonable request.
